# Comparative characteristics of laryngeal-resident mesenchymal stromal cell populations isolated from distinct sites in the rat larynx

**DOI:** 10.1186/s13287-017-0650-y

**Published:** 2017-09-29

**Authors:** Songyi Lee, Yeseulmi Kim, Hyun-Soo Shin, Jae-Yol Lim

**Affiliations:** Department of Otorhinolaryngology, Gangnam Severance Hospital, Yonsei University College of Medicine, 211 Eonju-ro, Gangnam-gu, Seoul 06273 Republic of Korea

**Keywords:** Larynx, Vocal folds, Macula flava, Mesenchymal stromal cells, Multipotent stem cells

## Abstract

**Background:**

Although tissue-resident mesenchymal stromal cells (MSCs) in the larynx have been described, their distinct characteristics and roles have not been thoroughly explored. Therefore, we investigated stem cell characteristics and regenerative potentials of single clonal populations isolated from rat epiglottic mucosa (EM), lamina propria (LP), and macula flava (MF) to determine whether they comprised laryngeal tissue-resident stem cells.

**Methods:**

Single clonal laryngeal cells were isolated following microdissection of the EM, LP, and MF from the rat larynx. Several clonal populations from the three laryngeal subsites were selected and expanded in vitro. We compared the stem cell characteristics of self-renewal and differentiation potential, as well as the cell surface phenotypes and gene expression profiles, of laryngeal MSC-like cells to that of bone marrow MSCs (BM-MSCs). We also investigated the regenerative potential of the laryngeal cells in a radiation-induced laryngeal injury animal model.

**Results:**

Self-renewing, clonal cell populations were obtained from rat EM, LP, and MF. EM-derived and LP-derived clonal cells had fibroblast-like features, while MF-resident clonal cells had stellate cell morphology and lipid droplets containing vitamin A. All laryngeal clonal cell populations had MSC-like cell surface marker expression (CD29, CD44, CD73, and CD90) and the potential to differentiate into bone and cartilage cell lineages; EM-derived and MF-derived cells, but not LP-derived cells, were also able to differentiate into adipocytes. Clonal cells isolated from the laryngeal subsites exhibited differential extracellular matrix-related gene expression. We found that the mesenchymal and stellate cell-related genes desmin and nestin were enriched in laryngeal MSC-like cells relative to BM-MSCs (*P* < 0.001). Growth differentiation factor 3 (GDF3) and glial fibrillary acidic protein (GFAP) transcript and protein levels were higher in MF-derived cells than in other laryngeal populations (*P* < 0.001). At 4 weeks after transplantation, laryngeal MF-derived and EM-derived cells contributed to laryngeal epithelial and/or glandular regeneration in response to radiation injury.

**Conclusions:**

These results suggest that cell populations with MSC characteristics reside in the EM, LP, and MF of the larynx. Laryngeal MSC-like cells contribute to regeneration of the larynx following injury; further investigation is needed to clarify the differential roles of the populations in laryngeal tissue regeneration, as well as the clinical implications for the treatment of laryngeal disease.

**Electronic supplementary material:**

The online version of this article (doi:10.1186/s13287-017-0650-y) contains supplementary material, which is available to authorized users.

## Background

Damage to vocal folds (VFs), which induces disorganization of the layered ultrastructure of VFs, often leads to severe dysphonia. During normal phonation, the mucosal wave is vertically propagated by vibration of epithelial mucosa overlying the superficial lamina propria (LP) (Reinke’s space), which contains extracellular matrix (ECM) proteins that confer viscoelastic properties [1]. Changes in viscoelasticity due to disruption of the arrangement of ECM proteins impact voice quality. In many recent years, regenerative therapies have been developed to either repair or reconstitute ECM proteins by cell therapy, including transplant of exogenous stem cells or primary tissue-resident cells into injured VFs. However, the biological functions of primary VF-resident cells in VF homeostasis and regeneration following injury have not been well characterized. In order to establish the proof of concept for VF-derived cell-based therapies, the roles of tissue-resident cells and their mechanisms of action in VF must be elucidated.

The presence of a population of putative stem/progenitor cells in the VFs has been suggested by the detection of label-retaining cells and side population cells [2–4]. Mesenchymal stromal cell (MSC)-like cells have been isolated from the epiglottic mucosa (EM) of the canine larynx [5]. In addition, VF fibroblasts in the LP and stellate cells in the macula flava (MF) are tissue-resident cells known to regulate ECM metabolism and maintain VF homeostasis. Recently, multiple groups have assessed the stem cell characteristics of human LP fibroblasts and MF stellate cells, and proposed that they are VF-resident stem/progenitor cells [6, 7]. Although several populations from laryngeal tissues have been characterized as stem/progenitor cells, the exact localization of tissue-specific stem cells and their comparative characteristics have not yet been explored. Furthermore, cells obtained by adherence to plastic generally contain mixed populations, preventing a definitive conclusion about the stem cell characteristics of the populations. Purification of clonal cells is important not only for stem cell characterization, but also for the advancement of single-cell biology for translation in future clinical applications. We previously reported a subfractionation culture method that allowed us to obtain homogeneous, clonal MSCs from bone marrow, adipose tissue, and salivary glands [8–10]. In this study, using the clonal cell culture method, we isolated several self-renewing, clonal populations generated from single cells from the EM, LP, and MF of rat larynges. We compared their stem cell properties to those of representative MSCs from bone marrow (BM-MSCs). These populations all expressed MSC-like phenotypes and possessed the capacity to undergo differentiation into mesenchymal lineage cells. We then assessed the differential gene expression profiles of laryngeal MSC-like cells and, for the first time, found that expression of pluripotent stem cell marker, growth differentiation factor 3 (GDF3), is a distinguishing feature of MF-derived cells.

We also tried to further demonstrate that these specifically isolated single clonal cells actually have an effect on repairing and/or regenerating damaged laryngeal tissue, by using an irradiation-induced fibrosis animal model. Avoiding radiation exposure to the larynx is a significant clinical problem for patients with head and neck cancer, since irradiation-induced laryngeal fibrosis, edema, and desiccated mucosa result in intractable dysphonia [11–13]. The use of MSCs as a treatment to prevent or ameliorate damage associated with radiation may be a useful approach. For these reasons, we employed our previously established irradiation-induced laryngeal dysfunction animal model to investigate regenerative potential following radiation injury [13]. Results presented herein will advance the understanding of laryngeal tissue stem/progenitor cells, and serve as a prerequisite step for development of clinical VF regenerative therapies.

## Methods

### Animal experiments

Female Sprague–Dawley (SD) rats (6 weeks of age, 180–200 g) were purchased (Orient Bio, Gyeonggi-Do, Korea) and acclimatized at 20–26 °C to a 12-hour light cycle for 1 week before use. The study was approved by the Animal Ethics Committee of Inha University Hospital (Permit Number: INHA 141003-330-3). The regulations of the Institutional Animal Care and Use Committee of the Life Sciences Laboratory were followed for all animal experimental procedures. The minimum number of animals needed to achieve study objectives was chosen for ethical and economic reasons.

**Fig. 1:**
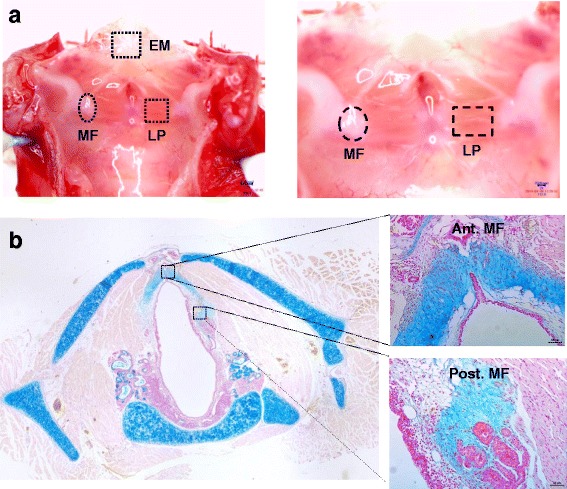
Location of laryngeal tissue-resident putative stem/progenitor cells. **a** Cross-sectional view of the rat larynx and the sites of dissection of epiglottic laryngeal epiglottic mucosa (EM), vocal fold (VF), lamina propria (LP), and macula flava (MF). Scale bars, 50 μm (left); 20 μm (right). **b** Alcian blue staining for identification of anterior (Ant. MF) and posterior MF (Post. MF) rat VFs. Scale bars, 50 μm. Whole larynx staining image generated by stitching together 40× microscopic images

The larynges of three SD rats were extracted and folded out to expose the internal structures. Laryngeal tissues were dissected from the EM, LP, and MF under a stereo microscope (Olympus, Tokyo, Japan) (Fig. 1a). EM tissue samples were obtained from the epiglottic mucosa, and LP tissue samples were acquired from the membranous VF. The MF is located at the anterior and posterior end of the VF, which is connected to thyroid cartilage (anterior MF) and arytenoid cartilage (posterior MF). The precise location of the MF was also visualized by Alcian blue (pH 2.5) staining (Fig. 1b). IMT i-Solution software (IMT i-Solutions, Vancouver, BC, Canada) was used for imaging, scanning, and processing.

### Isolation of laryngeal tissue-resident clonal cells from different laryngeal subsites

Each tissue sample was washed with 2% Antibiotic–Antimycotic solution (Gibco BRL, Gaithersburg, MD, USA) in Hank’s Balanced Salt Solution (HBSS; Gibco BRL). Samples were then treated with 0.05% collagenase B (Roche, Mannheim, Germany) and DNase I (Roche) for 1 hour at 37 °C. The enzymatic activity was stopped by adding Dulbecco’s Modified Eagle Medium (DMEM; Gibco BRL) containing 10% fetal bovine serum (FBS; Gibco BRL) and 1% Antibiotic–Antimycotic. The supernatant was removed by centrifugation at 470 rcf for 5 minutes, after which the pellet was resuspended in DMEM containing 10% FBS and 1% Antibiotic–Antimycotic, and then cultured at 37 °C under 5% CO_2_. When single cell-derived colonies were formed, each colony was isolated using a sterile 4.7 mm × 8 mm cloning cylinder (Bel-Art Products, Wayne, NJ, USA) and then transferred to a six-well plate (Falcon, Thermo Fisher Scientific, Waltham, MA, USA). The culture medium, low-glucose (1 g/L) DMEM (L-DMEM; Gibco BRL) containing 10% FBS and 1% Antibiotic–Antimycotic, was changed every 2–3 days. The same lot number of FBS products (Lot. No. 1831333; Gibco) was used for cell cultures. Cells were split when they were 70–80% confluent, and several clonal cells from the three laryngeal subsites were selected and expanded in vitro. A minimum of three clonal populations were established from each subsite, and at least three different clonal cells passaged fewer than 10 times were used in our experiments.

### Isolation of rat BM-MSCs

The femurs of SD rats were extracted and washed with HBSS containing 2% Antibiotic–Antimycotic. The proximal and distal areas of the femur were removed and the marrow was extracted by injecting DMEM in the femoral shaft. The extracted marrow was filtered through a 70-μm cell strainer (Falcon, Thermo Fisher Scientific) and centrifuged at 470 rcf for 5 minutes. The supernatant was removed and the pelleted bone marrow was cultured in DMEM with 10% FBS and 1% Antibiotic–Antimycotic at 37 °C under 5% CO_2_ for 24 hours. Floating hematopoietic cells were washed away with phosphate buffered saline (PBS; Gibco BRL), after which the medium was changed. Using sterile cloning cylinders, single colonies were isolated and transferred to six-well plates. The cells were cultured in L-DMEM containing 10% FBS and 1% Antibiotic–Antimycotic, which was changed every 2–3 days. Cells were split when they reached 70–80% confluency, and cells passaged fewer than 10 times were used in the experiments.

### Cell morphology and validation of MF stellate cells

We observed the morphology of the cells by phase-contrast microscopy. To confirm that a cell isolated from the MF was stellate, we assessed vitamin A autofluorescence using the UV filter of an Axiovert 200 inverted fluorescence microscope (Carl Zeiss, Oberkochen, Germany), and then conducted retinoid-based fluorescence-activated cell sorting (FACS). The cells were sorted on a FACSAria cell sorter (BD Biosciences, San Jose, CA, USA) using a 450/50-nm band-pass filter.

### Immunofluorescence staining

The cells were seeded on glass coverslips (Marienfeld-Superior, Baden-Württemberg, Germany) and cultured for 24 hours, then fixed with 4% paraformaldehyde (Wako, Osaka, Japan), washed with PBS, and permeabilized with 0.2% Triton X-100 (Sigma-Aldrich, St. Louis, MO, USA). Blocking was conducted for 1 hour using PBS containing 2% goat serum (Vector Laboratories, Burlingame, CA, USA) and 1% bovine serum albumin. Samples were then incubated with antibodies against vimentin (Cell Signaling Technology, Danvers, MA, USA), α-smooth muscle actin (α-SMA; Millipore, Darmstadt, Germany), glial fibrillary acidic protein (GFAP; Abcam, Cambridge, UK), and growth differentiation factor 3 (GDF3; Bioss, Woburn, MA, USA) at 4 °C overnight, after which they were washed three times with PBS. The samples were incubated with Alexa Fluor® 488-conjugated goat anti-rabbit and Alexa Fluor® 555-conjugated goat anti-rabbit secondary antibodies (Invitrogen, Carlsbad, CA, USA) for 1 hour at 37 °C, after which the cells were mounted with mounting solution containing 4′,6-diamidino-2-phenylindole dihydrochloride (DAPI; Vector Laboratories).

### Reverse transcription polymerase chain reaction and quantitative real-time PCR

We extracted RNA from the isolated cells using TRIzol® (Thermo Fisher Scientific), and then synthesized cDNA using the RNA as a template with a Reverse Transcription System Kit (Bioneer, Daejon, Korea) with incubations for 15 minutes at 37 °C, 5 seconds at 85 °C, and 10 minutes at 4 °C. PCR was conducted using gene-specific primers for the synthesized cDNA. The amplification step was repeated for 28 cycles consisting of 30 seconds of denaturation at 94 °C, 30 seconds of annealing at 60 °C, and 30 seconds of extension at 72 °C. After completion, the product was dyed with ethidium bromide (Thermo Fisher Scientific) and subjected to gel electrophoresis in 1.5% agarose.

The synthesized cDNA was included in PCR mixtures with SYBR® Green Premix (Takara Bio, Shiga, Japan), diethylpyrocarbonate-treated distilled water (Welgene, Daegu, Korea), and gene-specific primers. The reactions were conducted using a real-time PCR system (Applied Biosystems, Carlsbad, CA, USA), as follows: 94 °C for 10 minutes, followed by 40 cycles of 95 °C for 10 seconds, 55 °C for 25 seconds, and 72 °C for 30 seconds. Each PCR amplification was performed in triplicate wells. The results were normalized against those for the reference gene glyceraldehyde-3-phosphate dehydrogenase (GAPDH) by the 2^–ΔΔCtt^ method, and results are presented as the mean ± SD of real-time PCR triplicates. The primer sequences are presented in Table 1.

**Table 1:** Polymerase chain reaction primers

Primer		Sequence 5′ → 3′
Alpha actin, α-actin (*Acta1*)	Forward:	ATTGGTATGGAGTCCCGACCG
	Reverse:	TCCCAGTGTAAGGTAGCCG
Alpha smooth muscle actin, α-SMA (*Acta2*)	Forward:	GAGAGGGATCCTGACCTGA
	Reverse:	CCACGCGAAGCTCGTTATAG
CD133 (*Prom1*)	Forward:	GGTCAGCCTGCATTCGCTAA
	Reverse:	CTGGACCACGTTGAGGAAGA
Desmin (*Des*)	Forward:	CAACCTTCCGCTCCAGACCT
	Reverse:	TCATGTTGTTGCTGTGTGGC
Epidermal growth factor (*Egf*)	Forward:	GCCAATGCTCAGAAGGCTAC
	Reverse:	CGTAAGTCTCGGTGCTGACA
Fibronectin (*Fn*)	Forward:	AGACTGCAGTGACCACCATCC
	Reverse:	CAATGTGTCCTTGAGAGCATAGAC
Glyceraldehyde-3-phosphate dehydrogenase (*Gapdh*)	Forward:	ATTCTACCCACGGCAAGTTCACTGG
	Reverse:	AGGGGCGGAGATGATGACCC
Growth differentiation factor 3 (*Gdf3*)	Forward:	TGCACTCTCTTCATGCCTCC
	Reverse:	GAACAGTTGGTGACGATGGC
Glial fibrillary acidic protein (*Gfap*)	Forward:	GCTCAATGACCGCTTTGCTA
	Reverse:	TCTGCCTGGTAAACGTCAGC
Hyaluronan synthase 2 (*Has2*)	Forward:	CCAATGCAGTTTCGGTGATG
	Reverse:	ACTTGGACCGAGCCGTGTAT
Hepatocyte growth factor (*Hgf*)	Forward:	TCGAGCTATCGCGGTAAAGA
	Reverse:	TGTGATCCATGGGACCTCTG
Nestin (*Nes*)	Forward:	TGCTGATGAGGAAGGAGCAG
	Reverse:	AAGCCTCATCCCAGAGACC
Peroxisome proliferator-activated receptor gamma (*Pparg*)	Forward:	TTCGGAATCAGCTCTGTGGA
	Reverse:	CCATTGGGTCAGCTCTTGTG
Tropoelastin (*Eln*)	Forward:	AGAAGCCTCGACATTAGATTTGGT
	Reverse:	GGAGCTATTCCCAGTGTGAGAAGT
Type I collagen (*Col1a1*)	Forward:	TTGACCCTAACCAAGGATGC
	Reverse:	CACCCCTTCTGCGTTGTATT
Vascular endothelial growth factor (*Vegf*)	Forward:	AGGCTGCACCCACGACAGAA
	Reverse:	CTGGAAGATGTCCAGGG
Vimentin (*Vim*)	Forward:	GCCTATGTGACCCGGT
	Reverse:	AGACGTGCCAGAAAGCATTGTCAA
von Willebrand factor (*Vwf*)	Forward:	TGCTCTTACGCCCATCTCT
	Reverse:	CACTCATACTCTGGGCAGCA
Wingless-type MMTV integration site family, member 1 (*Wnt1*)	Forward:	CGTGAACATAGCCTCCTCCA
	Reverse:	AATTGCCACTTGCACTCTCG

### Western blot analysis

Protein samples were isolated from cell lysates (50 μg), and then mixed in reducing buffer, boiled, resolved by sodium dodecyl sulfate (SDS)-polyacrylamide gel electrophoresis, and transferred to a polyvinylidene difluoride (PVDF) membrane by electroblotting. The blot was incubated overnight at 4 °C in a blocking solution with primary antibodies directed to the following antigens: GFAP, peroxisome proliferator-activated receptor γ (PPARγ), Wnt1 (Abcam, Cambridge, UK), growth differentiation factor 3 (GDF3; Bioss, Woburn, MA, USA), Smad2/Smad3, phospho-Smad2/Smad3 (Cell Signaling Technology, Danvers, MA, USA), transforming growth factor beta (TGF-β; Abcam, Cambridge, UK), and β-actin (Santa Cruz Biotechnology, Santa Cruz, CA, USA). After the blots were washed with 0.1% Tween® 20 (Sigma-Aldrich) in 1 × PBS, they were incubated with horseradish peroxidase-conjugated secondary antibodies corresponding to each primary antibody, then analyzed by enhanced chemiluminescence detection (GE Healthcare Life Sciences, Pittsburgh, PA, USA). Quantification of protein band intensities in three independent experiments was performed, and the relative ratio under the constant reference β-actin was calculated.

### Cell proliferation assay

For cell proliferation analysis, 2 × 10^4^ cells were seeded in a 100-mm culture dish. Cells were detached by treatment with 0.05% trypsin/ethylenediaminetetraacetic acid (Gibco BRL) every 3 days. Live cells were counted by the trypan blue exclusion method, which utilizes characteristics that have an intact cell membrane excluding certain trypan blue dye. Briefly, a 10 μl mixture of cell suspended in growth media and trypan blue solution was inserted between the hemocytometer and the cover slip, and the number of unstained cells was then counted. We plotted cell proliferation based on the number of live cells over 7–20 passages, and calculated the doubling time. At least three independent experiments were performed.

### Phenotypic analysis by flow cytometry

Cells isolated at passage 6 or 7 were resuspended in PBS, and then incubated with antibodies conjugated to fluorescein isothiocyanate (FITC) or phycoerythrin (PE) at 4 °C for 30 minutes. Cells were analyzed using a FACSCalibur system (BD Biosciences) and data were analyzed using CellQuest™ (BD Biosciences). Dead cells were excluded by forward scatter (FSC)/side scatter (SSC) parameters, and viable cells were further gated as a singlet population. Antibodies against CD29, CD90, CD73, CD31, CD45, nestin (BD Biosciences), CD34, CD105 (Abcam), and CD44 (R&D Systems, Minneapolis, MN, USA) were used in conjunction with appropriate isotype controls (BD Biosciences). Isotype-matched control antibodies were used in each antibody analysis.

### Multilineage differentiation

For adipogenic differentiation, 3 × 10^4^ cells were seeded in four-well plates (SPL, Gyeonggi-do, Korea) and incubated for 48 hours, after which they were washed twice with PBS and plated in adipocyte differentiation induction medium. The adipocyte differentiation induction medium contained 10% FBS (Gibco BRL), 2% penicillin/streptomycin (P/S; Gibco BRL), 10^–7^ M dexamethasone (DEX; Sigma-Aldrich), 0.5 mM isobutylmethylxanthine (Sigma-Aldrich), 10 μg/ml insulin (Sigma-Aldrich), and 100 or 200 μM indomethacin (Sigma-Aldrich) in high-glucose DMEM (Gibco BRL). The medium was changed every 2–3 days over the course of differentiation for 14 or 30 days. Differentiation was assessed by dyeing the cells with Oil Red O (Sigma-Aldrich).

For osteogenic differentiation, 3 × 10^4^ cells were seeded in a collagen-coated (Roche) four-well plate and cultured at 37 °C under 5% CO_2_. The osteogenic differentiation medium contained 10% FBS, 2% P/S, 10^–8^ M DEX, 10 mM β-glycerolphosphate (Sigma-Aldrich), 50 μg/ml ascorbic acid (Sigma-Aldrich), and 100 μM dibutyryl-cyclic AMP (Sigma-Aldrich) in Minimum Essential Medium Eagle, alpha modification (α-MEM; Welgene). The medium was changed every 2–3 days for 21 days. We dyed the cultures with 0.1% Alizarin Red S (pH 4.2) (Sigma-Aldrich) to confirm calcium tubercle formation.

For chondrogenic differentiation, 2 × 10^5^ cells were resuspended in a 15-ml conical tube and centrifuged at 840 rcf for 10 minutes. The samples were then cultured at 37 °C under 5% CO_2_. The chondrogenic differentiation induction medium contained 10% FBS, 2% P/S, 10^–7^ M DEX, 0.1% ITS premix (BD Bioscience), 50 μg/ml ascorbic acid, 40 μg/ml l-proline (Sigma-Aldrich), 10 ng/ml transforming growth factor (TGF)-β1 (R&D Systems), and 10 ng/ml TGF-β3 (R&D Systems) in α-MEM. The medium was changed to chondrogenic differentiation induction medium after 24 hours, and then changed every 2–3 days for 21 days. Upon completion of differentiation, pellets were embedded in optimum cutting temperature (OCT) compound, after which the 8-μm-thick frozen sections were dyed with Safranin O (Thermo Fisher Scientific). Cells passaged fewer than seven times were cultured individually and used for differentiation induction.

### In-vivo experiments

#### Irradiation and transplantation of EM, LP, and MF clonal cells

Twenty-five SD rats (6 weeks old, 150–200 g) were randomly divided into five groups (*n* = 5 per group) as follows: normal control (Normal), RT control (RT + Vehicle group), EM cell injection after irradiation (RT + EM group), LP cell injection after irradiation (RT + LP group), and MF cell injection after irradiation (RT + MF group). The rats locally irradiated in the laryngeal region, while the rest of the body was shielded from radiation. Prior to irradiation, rats were anesthetized using xylazine (10 mg/kg) and ketamine (110 mg/kg). Animals were firmly fixed in a plastic mold and irradiated with a 9-MeV electron beam from a linear accelerator (Clinac® iX; Varian Medical System, Palo Alto, CA, USA) using a single dose of 15 Gy. Rats that did not receive irradiation served as the control group.

After 4 weeks of irradiation, rats were anesthetized and the larynx was exposed using a custom-made laryngoscope and a surgical operating microscope (Carl Zeiss, Welwyn Garden City, UK). Vehicle or EM, LP, or MF cells labeled with the red fluorescent dye PKH26 (Sigma-Aldrich) were injected (1 × 10^5^ in 10 μl of PBS) into the unilateral VFs of the rats using a syringe with a 25-gauge needle.

#### Histological and immunofluorescence examination

At 4 weeks after transplantation, the larynges were removed post euthanasia for detection of the red fluorescent-labeled EM-derived, LP-derived, and MF-derived cells transplanted into irradiated rat VFs. The larynges were fixed with 4% paraformaldehyde, embedded in OCT compound (Sakura Finetek USA), and cut into 7-μm-thick sections. The slides were washed in PBS and stained with DAPI (Invitrogen) for 3–5 minutes to stain the cell nuclei. The slides were visualized using a confocal laser scanning microscope (Olympus).

For evaluation of histological changes, larynges were fixed and paraffin-embedded, and then sectioned into 4-μm slices. The sections were stained with hematoxylin and eosin (HE) and Masson’s trichrome (MTC), using standard pathology department protocols. For immunofluorescence, paraffin sections were deparaffinized in xylene, rehydrated in ethanol, and washed with PBS. Nonspecific binding was blocked with PBS containing 1% bovine serum albumin. Slides were incubated with primary antibodies for collagen type I (ABBiotec, San Diego, CA, USA), E-cadherin (R&D Systems), and aquaporin 4 (AQP4) and aquaporin 5 (AQP5) (Santa Cruz Biotechnology, CA, USA) overnight at 4 °C. After washing in PBS, slides were incubated with Alexa Fluor® 488-conjugated anti-rabbit IgG (Invitrogen), anti-goat secondary antibodies (Invitrogen), and Alexa Fluor® 594-conjugated anti-mouse and anti-goat secondary antibodies (Invitrogen) for 1 hour in the dark at 20–25 °C. The nuclei were counterstained with DAPI (Vector Labs) and then the slides were viewed using a confocal laser scanning microscope (Olympus). Fluorescence intensity was measured using ImageJ software.

### Statistics

Statistical analysis was conducted using the GraphPad Prism 5 package (GraphPad Software, La Jolla, CA, USA). One-way analysis of variance (ANOVA) and Tukey’s post-hoc test were used to determine intergroup differences. Differences of *P* < 0.05 were considered statistically significant.

## Results

### Isolation of laryngeal tissue-resident clonal cells from rat larynx

**Fig. 2:**
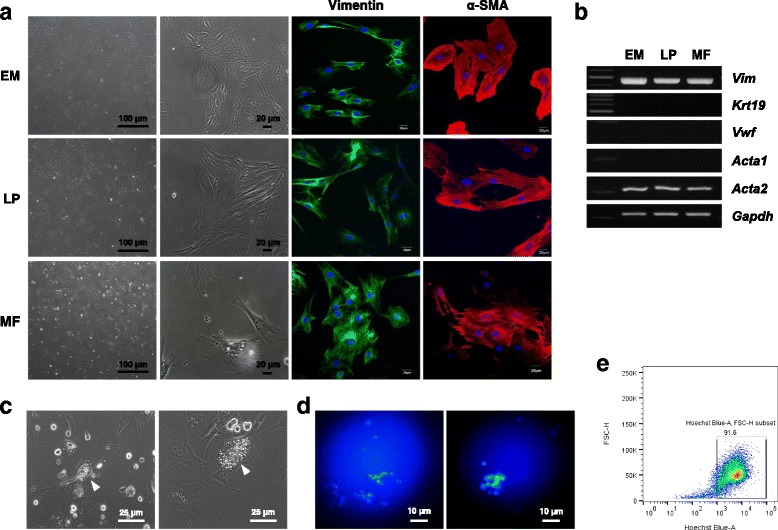
Isolation and validation of laryngeal tissue-resident clonal cells. **a** Clonal cells purified from laryngeal subsites of EM, LP and MF visualized by phase-contrast microscopy. Scale bars, 100 and 20 μm. Representative immunofluorescence staining images of laryngeal clonal cells expressing mesenchymal markers vimentin and α-SMA. **b** Expression of the genes *Krt19* (epithelial cell marker) and *Vwf* (endothelial cell marker) was not detected, whereas that of *Vim* and *Acta2* was detected in all laryngeal clonal cells. **c** MF stellate cells were validated to contain lipid droplets (arrow) by phase-contrast microscopy. Scale bars, 25 μm. These cells demonstrated vitamin A (retinoid) autofluorescence as assessed by **d** fluorescence microscopy (scale bars, 10 μm) and **e** retinoid-based FACS sorting. *EM* epiglottic mucosa, *LP* lamina propria, *MF* macula flava, *α-SMA* α-smooth muscle actin, *FSC* forward scatter

We employed the single clonal cell culture method to isolate several single clonal cells from microscopically dissected EM from larynges, and LP and MF from VFs, in three rats. After enrichment of clonal cells, at least three subpopulations isolated from various sites were selected and characterized. During subculture, the clonal cells isolated from the EM and LP had a spindle-shaped fibroblast-like appearance; however, clonal cells from the MF had a distinct star-like appearance (Fig. 2a). Immunofluorescence staining revealed that all clonal cells expressed the mesenchymal cell markers vimentin and α-SMA (Fig. 2a). RT-PCR confirmed that all clonal cells were purified mesenchymal cells that lacked epithelial (*Krt19*) or endothelial (*Vwf*) gene expression (Fig. 2b).

MF stellate cells were validated using a phase-contrast microscope. We found lipid droplets in the cytoplasm of clonal MF cells, which were absent in LP-derived and EM-derived cells (Fig. 2a, c). We also observed vitamin A autofluorescence in clonally expanded MF cells (Fig. 2d). We further confirmed vitamin A storage in single clonal MF cells by retinoid-based FACS sorting (Fig. 2e).

### Self-renewal capacity of laryngeal tissue-resident clonal cells

**Fig. 3:**
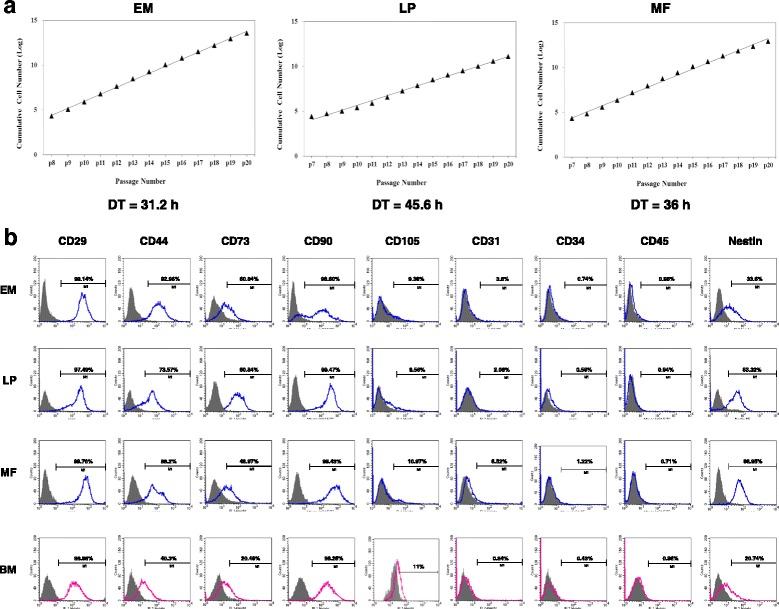
Clonal cell growth and surface marker expression profiles of laryngeal clonal cells. **a** Laryngeal tissue-resident cells from EM, LP, and MF displayed high proliferative activities up to passage 20, with doubling times (DT) of 31.2, 45.6, and 36 hours, respectively. **b** Flow cytometric analysis revealed that all laryngeal clonal cells expressed MSC markers CD29, CD44, CD73, and CD90, in the absence of CD105, CD31, CD34, and CD45. In addition, they were positive for nestin, a marker of undifferentiated stem cells. Experiments were performed in three biological replicates (at least three clonal populations) with similar results (data not shown). *EM* epiglottic mucosa, *LP* lamina propria, *MF* macula flava, *BM* bone marrow

The self-renewal properties of laryngeal-resident clonal populations were evaluated by long-term in-vitro proliferative activity. We cultured three clonal populations derived from the EM, LP, and MF up to passage 20 without obvious morphological changes during cultivation. We determined the rate of cell proliferation by calculating the doubling time during subculture. The population doubling time was 31.2, 45.6, and 36 hours for cells from the EM, LP, and MF, respectively (Fig. 3a). These results suggest that the isolated clonal populations are highly proliferative rather than dormant or quiescent.

### Characterization of MSC properties

#### MSC surface marker analysis

We performed flow cytometry to compare laryngeal MSC surface marker expression with BM-MSC marker expression. Laryngeal clonal cells expressed MSC markers such as CD29, CD44, CD73, and CD90, in the absence of expression of hematopoietic markers such as CD31, CD34, and CD45 (Fig. 3b). The MSC marker CD105 (endoglin) was not detected in laryngeal cells, although it was detected in BM-MSCs. In addition, nestin, a marker of undifferentiated stem cells, was observed in laryngeal clonal cells.

#### Mesenchymal lineage differentiation potential

**Fig. 4:**
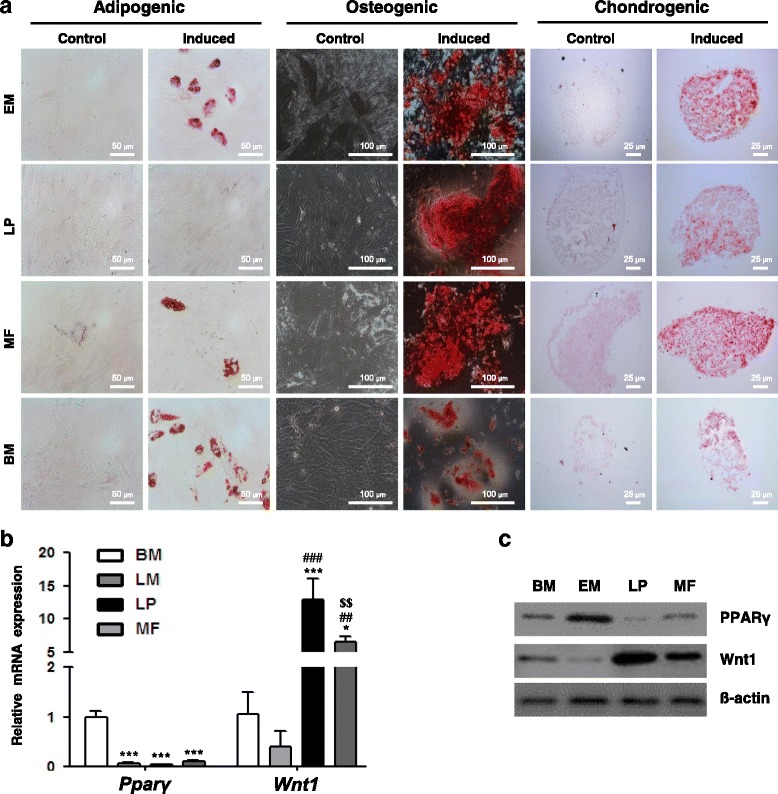
Differentiation potential into mesenchymal lineage cells. **a** Potential of clonal cells to differentiate into adipogenic, osteogenic, and chondrogenic lineage cells analyzed via lineage-specific cytochemical staining. Adipogenic differentiation was assessed by Oil Red O staining of intracellular lipid vacuoles. Indomethacin was added to induce adipogenic differentiation of MF cells. Scale bars, 50 μm. Osteogenic differentiation was assessed by Alizarin Red S staining of mineralized nodules. Scale bars, 100 μm. Chondrogenic differentiation was assessed by Safranin O staining of proteoglycans. Scale bars, 25 μm. Levels of PPARγ and Wnt1, which were related to the plasticity of laryngeal clonal cells, confirmed by **b** qRT-PCR and **c** Western blot analysis. Error bars, SD (*n* = 3 wells). *P < 0.05, ***P < 0.001 compared with BM-MSCs; ^##^ *P* < 0.01, ^###^ *P* < 0.001 compared with EM cells; ^$$^ *P* < 0.01 compared with LP cells. Refer to Additional file 1 for complete statistical values. *EM* epiglottic mucosa, *LP* lamina propria, *MF* macula flava, *BM* bone marrow

To determine their mesenchymal differentiation potential, we examined the ability of clonal cells to differentiate into adipogenic, osteogenic, and chondrogenic lineages upon appropriate induction (Fig. 4a). We cultured clonal cells in adipocyte differentiation-inducing media; both BM-MSCs and EM-derived clonal cells differentiated into adipocytes containing lipid vacuoles that stained with Oil Red O, while LP-derived and MF-derived clonal cells did not. However, when we increased the dose of indomethacin in the adipogenic differentiation medium to 200 μM, MF-derived cells, but not LP-derived cells, were induced to differentiate into adipocytes (Fig. 4a).

We performed Alizarin Red S staining to assess the formation of mineralized nodules in the osteogenic differentiation cultures. All laryngeal clonal cells and BM-MSCs differentiated and formed mineralized nodules after 21 days of osteogenic differentiation induction (Fig. 4a). Under chondrogenic differentiation conditions, all laryngeal clonal cells and BM-MSCs differentiated into chondrocytes (Fig. 4a). We confirmed the formation of proteoglycan using Safranin O dye 21 days after induction. Taken together, these data indicate that all laryngeal clonal cells possess MSC characteristics.

#### Role of Wnt signaling in laryngeal MSC-like cell plasticity

In addition, we explored the involvement of Wnt signals in the adipogenic differentiation potential of laryngeal MSC-like cells. We detected significantly higher Wnt1 mRNA and protein levels in LP and MF-derived cells compared to BM-MSCs and EM-derived cells, and lower PPARγ levels compared to BM-MSCs (Fig. 4b, c). These results suggest that Wnt signaling may regulate laryngeal MSC-like cell plasticity in undifferentiated LP-derived and MF-derived cells through inhibition of PPARγ expression. For more details of the statistical analysis, refer to Additional file 1.

### Molecular characteristics and biological functions of laryngeal-resident stem cells

**Fig. 5:**
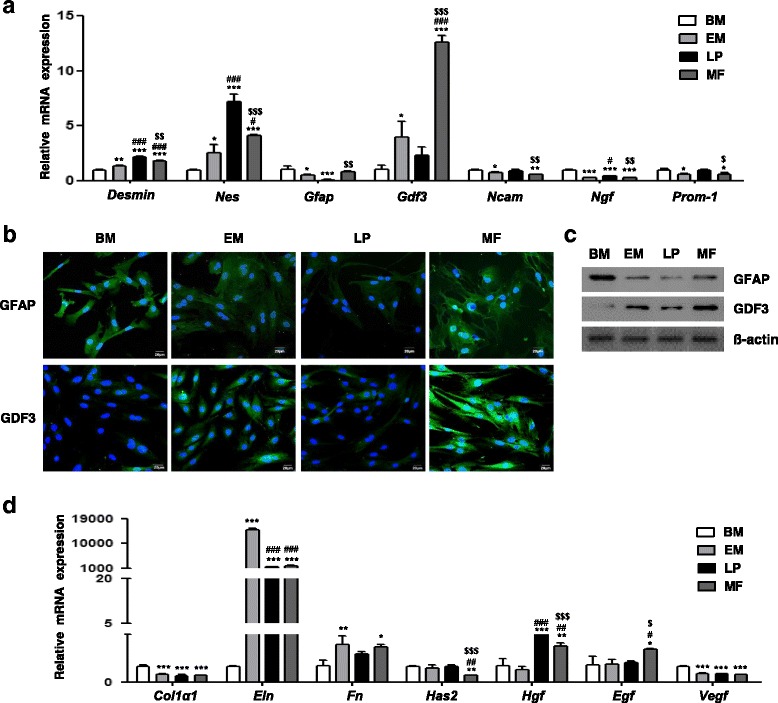
Molecular characteristics and biological functions of laryngeal clonal cells. **a** Gene expression of stellate cell markers *Des*, *Nes*, *Gfap*, *Gdf3*, *Ncam*, *Ngf*, and *Prom1* analyzed by qRT-PCR. Error bars, SD (*n* = 3 wells). *P < 0.05, **P < 0.01, ***P < 0.001 compared with BM-MSCs; ^#^ *P* < 0.05, ^###^ *P* < 0.001 compared with EM cells; ^$^ *P* < 0.05, ^$$^ *P* < 0.01, ^$$$^ *P* < 0.001 compared with LP cells. **b** Immunofluorescent staining was performed to identify differential markers of MF cells, GFAP and GDF3. Scale bars, 20 μm. **c** Protein expression of GFAP and GDF3 confirmed by western blot analysis. **d** Expression of ECM-related genes (*Col1a1*, *Eln*, *Fn*, and *Has2*) and growth factors (*Hgf*, *Egf*, and *Vegf*) analyzed in clonally expanded laryngeal MSC-like cells by qRT-PCR. Error bars, SD (*n* = 3 wells). *P < 0.05, **P < 0.01, ***P < 0.001 compared with BM-MSCs; ^#^ *P* < 0.05, ^##^ *P* < 0.01, ^###^ *P* < 0.001 compared with EM cells; ^$^ *P* < 0.05, ^$$$^ *P* < 0.001 compared with LP cells. Refer to Additional file 1 for complete statistical values. *EM* epiglottic mucosa, *LP* lamina propria, *MF* macula flava, *BM* bone marrow

We next performed qRT-PCR to further investigate the molecular characteristics of laryngeal-resident MSC-like cells and their potential biological roles in ECM regulation. We analyzed the relative mRNA levels of the stellate or stem cell markers desmin, nestin, GFAP, GDF3, neural cell adhesion molecule (NCAM), nerve growth factor (NGF), and CD133 (Prom1); the ECM-related markers collagen I, elastin, fibronectin, and hyaluronic acid; and the growth factors hepatocyte growth factor (HGF), epidermal growth factor (EGF), and vascular endothelial growth factor (VEGF) (Fig. 5).

We quantitatively analyzed the expression levels of stellate cell-related markers (Fig. 5a and Additional file 1). Laryngeal clonal cells expressed higher levels of desmin (*Des*) and nestin (*Nes*) than BM-MSCs. Within the laryngeal clonal MSC-like cell populations, the expression levels of *Des* and *Nes* were highest in LP-derived cells. The mRNA level of *Gdf3* was significantly higher in MF-derived cells than in other laryngeal clonal cells and BM-MSCs. Although we did not detect a statistical difference in *Gfap* mRNA expression in laryngeal MSC-like cells and BM-MSCs, the levels of *Gdf3* were higher in MF-derived cells than LP-derived cells, indicating GFAP and GDF3 may be distinguishing markers of MF-derived cells (Fig. 5b, c). However, NCAM (*Ncam*)*,* NGF (*Ngf*), and CD133 (*Prom1*) gene expression was lower in MF-derived cells compared to BM-MSCs and EM cells.

We detected downregulation of the ECM genes *Col1a1* (collagen I) in all laryngeal MSC-like cells and *Has2* (HA synthase) in MF-derived cells, while *Eln* (elastin) and *Fn1* (fibronectin) were upregulated in EM-derived and MF-derived cells relative to BM-MSCs (Fig. 5d and Additional file 1). EM-derived cells expressed a much higher level of *Eln*, while the MF-derived cells had much lower *Has2* expression, than other laryngeal MSC-like cells. The mRNA level of *Hgf* was elevated in the LP-derived and MF-derived cells relative to BM-MSCs and EM-derived cells. MF-derived cells exhibited a higher level of EGF than other laryngeal MSC-like cells. All laryngeal resident MSC-like cells showed decreased levels of VEGF.

### Regenerative potential of laryngeal clonal stem cells

**Fig. 6:**
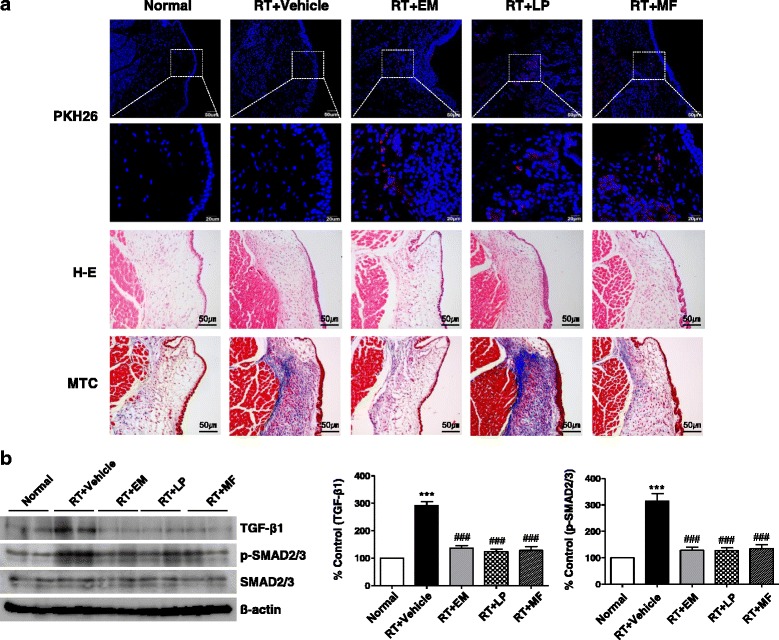
Engraftment of transplanted laryngeal clonal cells and their roles in irradiation-induced fibrosis or inflammation. **a** Red fluorescent dye PKH26-labeled laryngeal MSCs were observed in the irradiated VF site. Representative image of HE staining and MTC staining at 4 weeks post transplantation. At least three random tissue sections per VF from five rats were chosen for evaluation. Scale bars, 20 or 50 μm. **b** Western blot analysis was performed to detect expression of TGF-β1, SMAD2/3, and p-SMAD2/3 in rat VFs. Data representative of three independent experiments. One-way ANOVA, Tukey’s post-hoc test. ***P < 0.001 vs Normal; ###*P* < 0.001 vs Vehicle. Refer to Additional file 1 for complete statistical values. *MSC* mesenchymal stromal cells, *VF* vocal fold, *RT* radiation, *EM* epiglottic mucosa, *LP* lamina propria, *MF* macula flava

To determine whether laryngeal clonal MSC-like cells possessed regenerative potential, we transplanted them into the VFs of rats following radiation damage to the larynx. We performed histological evaluations of ECM production, as well as epithelial regeneration and laryngeal hydration. We observed a few PKH26-labeled EM-derived, LP-derived, and MF-derived cells at the site of injection in rat larynges 4 weeks post transplantation. Histological analysis of HE and MTC staining revealed that the epithelial thickening and subepithelial fibrosis that occurred after irradiation were alleviated in the groups transplanted with EM-derived and MF-derived cells (Fig. 6a). The expression of TGF-β1 and phosphorylated SMAD2/3 (p-SMAD2/3) in rat VFs after transplantation of laryngeal clonal MSC-like cells was attenuated, while laryngeal irradiation led to a significant upregulation of p-Smad2/3 and TGFβ1 (Fig. 6b and Additional file 1).

**Fig. 7:**
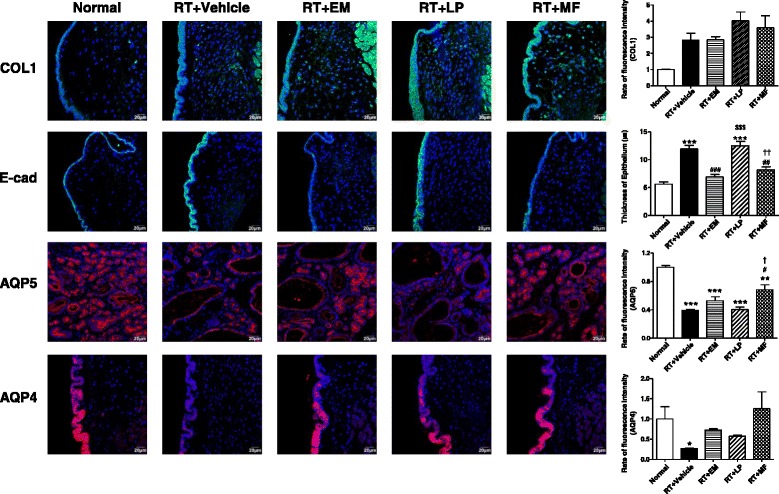
Laryngeal regeneration after transplantation of laryngeal clonal cells following radiation damage. Immunofluorescent staining for collagen I, E-cadherin, AQP4, and AQP5 performed on irradiated rat VFs. At least three random tissue sections per VF from five rats were chosen for evaluation. Scale bars, 20 μm. Fluorescence intensity and epithelial thickness measured using ImageJ. Error bars, SD (*n* = 5 animals). One-way ANOVA, Tukey’s post-hoc test. *P < 0.05, **P < 0.01, ***P < 0.001 vs Normal; ^##^ *P* < 0.01, ^###^ *P* < 0.001 vs Vehicle; ^$$$^ *P* < 0.001 vs EM; ^†^ *P* < 0.05, ^††^ *P* < 0.01 vs LP. Refer to Additional file 1 for complete statistical values. *RT* radiation, *EM* epiglottic mucosa, *LP* lamina propria, *MF* macula flava, *COL1* collagen I, *E-cad* Ecadherin, *AQP* aquaporin

Immunofluorescence analysis of VFs revealed increased production of collagen I in the irradiated and vehicle (PBS)-injected VFs, which was not reduced in all treated VFs. Expression of the epithelial junctional adherence marker E-cadherin was increased in the Vehicle group, suggesting thickening of the epithelium compared to the nonirradiated control group. In the groups transplanted with EM-derived and MF-derived cells, but not LP-derived cells, the epithelial thickening was significantly alleviated. After irradiation, the expression of aquaporins (AQPs), which are involved in VF epithelial hydration and lubrication, was decreased in VFs (AQP4) and laryngeal glands (AQP5). The expression of AQP5 was significantly restored after MF cell injection and, although the trend was not significant, the expression of AQP4 was also increased (Fig. 7 and Additional file 1).

## Discussion

We isolated clonal populations from different laryngeal subsites, including the EM of the larynges, and the LP and MF of the VFs, and demonstrated that these clonal cells represent laryngeal tissue-resident MSC-like cells. These highly proliferating clonal populations maintained their MSC surface marker expression and their potential to differentiate into mesenchymal lineage cells during cultivation in vitro. These results support the existence of laryngeal tissue-resident cells, such as VF fibroblasts and MF stellate cells, that share MSC properties and may participate in repopulation of laryngeal cells or contribute to laryngeal homeostasis; it is not yet clear how many populations are present in the quiescent state or how they are influenced by microenvironmental injury to participate in the regenerative process.

Laryngeal tissue-resident stem/progenitor cells are promising candidates for cell-based regenerative medicine to improve various voice disorders induced by scarring, radiation damage, or senescence. The nomenclature for VF-resident cells has been determined by cell morphology. Fibroblast-like cells in the LP and star-like cells in the MF have been named VF fibroblasts and MF stellate cells, respectively. Recent studies have shown that activated VF fibroblasts have therapeutic potential for the repair of VF scarring through ECM modulation [14, 15]. Another recent study by Thibeault et al. suggested that VF fibroblasts are MSCs which reside in the LP of VFs [6]. In addition, the paracrine release of inflammatory cytokines or ECM-regulating proteins by VF fibroblasts supports their potential therapeutic role in VF remodeling [16–18]. Another population of VF-resident cells in the MF (MF stellate cells) has long been known to be involved in the metabolism of the ECM of VFs [19–21]. Sato et al. [7] recently evaluated the characteristics of MF stellate cells and proposed that they are MSCs residing in the stem cell niche-like MF.

Although stem cell characteristics of laryngeal-resident cells have been investigated, biological functions of tissue-resident stem/progenitor cells from different sources in laryngeal regeneration and tissue homeostasis are still not well understood. In this study, we isolated single clonal cells from three different subsites to explore the stem cell characteristics of laryngeal tissue-specific stem/progenitor cells, using our method of subfractionation culture to enable isolation of single clone-derived homogeneous stem cell populations [10]. When compared with BM-MSCs, our results revealed that VF fibroblasts, MF stellate cells, and EM fibroblasts all display self-renewal capacity and multipotent stem cell features consistent with the presence of laryngeal tissue-resident MSC-like cells. Immunophenotypic analysis by flow cytometry demonstrated that the clonal cells from the three subsites expressed MSC surface markers CD29, CD44, CD73, and CD90, but not CD31, CD34, and CD45, similar to BM-MSCs. The expression of CD105 was not observed on laryngeal cells. Although Dominici et al. [22] proposed “standard criteria” for defining MSCs, the criteria do not include all sources of MSCs. MSC-like cells, which share MSC characteristics but are distinct in their surface marker expression and differentiation, have been isolated from various sources [23]. For example, CD105 expression has been reported to be variable and occasionally absent in tissue-resident or circulating MSCs [23, 24]. In addition, we observed synthesis of nestin, which is elevated during activation of somatic stem cells, in laryngeal clonal cells, suggesting that these clonal cells represent undifferentiated stem/progenitor cells [25]. Similar to BM-MSCs, laryngeal clonal cells possessed multipotent mesodermal differentiation capacity. Collectively, the laryngeal clonal cells isolated from rat EM, LP, and MF possessed MSC-like properties, but it remains to be determined whether these clonal populations represent distinct MSC subpopulations.

We observed plasticity in the potential of laryngeal MSC-like cells to differentiate into adipocytes and osteoblasts. LP-derived and MF-derived cells had lower adipocyte differentiation potential than BM-MSCs, but better osteoblast differentiation potential. This difference may be related to differential Wnt signaling in the subsets. Wnt is known to inhibit differentiation into adipocytes and induce differentiation into osteoblasts [26]. Analysis of gene expression revealed that Wnt1 gene expression levels were significantly higher in LP-derived and MF-derived cells than in EM-derived cells and BM-MSCs. Wnt1 inhibits adipocyte differentiation by suppressing expression of PPARγ [27]. LP-derived and MF-derived cells with high levels of Wnt1 also had very low expression of PPARγ, which is essential for adipogenesis and the induction of lipid production [28]. MF-derived cells, which had relatively low Wnt1 expression compared to LP cells, could be induced to differentiate into adipocytes in media containing twice the standard concentration of indomethacin. Indomethacin induces adipocyte differentiation by inhibiting Wnt/β-catenin signaling [29]. These results demonstrate that the expression of Wnt affects the differentiation potential of laryngeal MSC-like cells.

In this study, we isolated MF stellate cells from rat VFs. Stellate cells have been shown to exist in human liver, pancreas, lungs, kidneys, spleen, and ovaries. They are generally star-shaped, but may show various morphologies depending on their location and activation status [30, 31]. Stellate cell activation is associated with loss of cytoplasmic vitamin A (retinol) stores, and detection of vitamin A in lipid droplets has been widely applied to validate the identity of stellate cells [32]. Furthermore, their specific autofluorescence can be used for stellate cell separation and purification. In this study, we performed retinol-based FACS to assess whether isolated MF cells are stellate cells containing vitamin A.

Stellate cells are known to express various intermediate filaments, including desmin and GFAP, as well as the neural stem cell marker nestin. In addition, neurotrophins, such as NGF and NCAM, and the liver stem/progenitor cell marker CD133 (Prom1) have been reported to be stellate cell markers [33, 34]. However, in this study, the mRNA levels of desmin, nestin, NGF, and CD133 were highest in LP-derived cells among all laryngeal clonal populations. GFAP gene expression was only elevated in MF cells compared to other laryngeal clonal cells, although it was not significantly different from expression in BM-MSCs. These results suggest that the existing stellate cell-related markers cannot be applied uniformly to laryngeal stem cells, especially MF stellate cells. To identify another specific marker of MF-derived cells, we searched for proteins highly expressed in preadipocytes, based on the fact that stellate cells, like preadipocytes, contain lipid droplets. We found that expression of the pluripotent stem cell gene *GDF3*, which inhibits adipocyte development and is highly expressed in preadipocytes [35, 36], was highly expressed in MF-derived cells compared to BM-MSCs and other laryngeal MSC-like cells. We also observed robust GDF3 protein expression in MF-derived cells through immunofluorescence staining and western blot analysis. Taken together, such findings suggest that GDF3 can be used as a new marker for MF stellate cells, allowing MF-derived cells to be distinguished from other laryngeal MSC-like cells. Further investigations are warranted to better understand the behavior of human laryngeal stem cells, although we have found that human LP-derived or MF-derived cells show similar phenotypic expression (data not shown).

Various growth factors and cytokines secreted by stem cells play important roles in tissue repair; thus, understanding the mechanism of ECM modulation is important [37, 38]. In this study, we compared the ECM expression characteristics of laryngeal clonal MSC-like cells with those of BM-MSCs, which have been widely used for tissue engineering [39, 40]. When compared to BM-MSCs, LP-derived and MF-derived cells had significantly higher expression of HGF, a factor that promotes wound healing, inhibits collagen deposition, reduces scar formation, and prevents fibrosis [41]. MF-derived cells expressed high levels of EGF mRNA relative to other laryngeal MSC-like cells and BM-MSCs. EGF promotes cell growth, differentiation, and division, and quickly repairs damage in epithelial tissues [42]. These findings suggest that LP-derived cells and MF-derived cells may be more closely related to ECM regulation in LP and epithelial regeneration of VFs, respectively. Interestingly, all laryngeal MSC-like cells expressed low levels of VEGF, which may be attributed to the scant vascular structure of VFs [43].

The mRNA levels of *Col1a1* in laryngeal MSC-like cells and *Has2* in MF cells were 0.5 times lower than in BM-MSCs. However, fibronectin and elastin were more highly expressed in laryngeal MSC-like cells than in BM-MSCs. In addition, tropoelastin expression was more than 1500 times higher in all laryngeal clonal MSC-like cells than in BM-MSCs. Further investigation into ECM synthesis by laryngeal clonal MSC-like cells is required to enable the development of cell therapies for larynx repair and function recovery. The synthesis of various cytokines and growth factors by laryngeal MSC-like cells, as well as their interaction with innate neighboring cells, may be essential to their contribution to the wound healing process.

In this study, PKH26-labeled laryngeal MSCs appeared to be distributed in the irradiated VFs for up to 1 month after allogeneic transplantation. The survival of laryngeal MSCs in the injured VFs was probably related to the characteristics of MSCs, as well as tissue properties of VF [44]. MSCs have immunosuppressive features by themselves, which allow transplanted allogeneic MSCs to survive in recipient tissues. Nevertheless, an autotransplantation model using autologous MSCs for implantation into damaged VF may provide better results on VF regeneration following injury. Moreover, the number of tissue samples might be insufficient to obtain adequate statistical power, and additional investigations are required to confirm our speculations.

MF-derived cells contributed to laryngeal epithelial and glandular regeneration following radiation injury, but LP-derived cells were unable to remodel the VF after radiation in rats. In addition, EM-derived cells possessed regenerative potential for laryngeal epithelium, but they did not affect the level of AQP expression in the epithelium or laryngeal glands. Other stellate cell systems in the liver and pancreas have been suggested to be tissue stem cells that contribute to regeneration of injured organs through differentiation [45, 46]. In addition to tissue-resident stem cells, alternative cell sources for cell therapy, including migrating or transplanted MSCs from other organs (e.g., BM or adipose tissue), have been studied to determine whether they contribute to VF regeneration after injury, either by differentiation into VF-resident cells or by paracrine secretion of factors [44, 47, 48]. Although we did not track the cells from bone marrow to home into injured VFs, the current literature suggests that MSC-like cells, regardless of cell source, may promote VF wound repair or regeneration.

## Conclusions

In summary, we isolated and characterized laryngeal tissue-resident clonal cells from the EM, LP, and MF of rat larynges. The laryngeal cells possessed self-renewal and MSC surface marker expression, and the capacity to differentiate into mesenchymal lineage cells. The expression of markers such as desmin, nestin, GDF3, and GFAP varied with cell type. The synthesis of growth factors such as HGF and EGF indicated that the cells may have ECM-regulating biological functions. We also found that laryngeal MSC-like cells may play a crucial role in laryngeal regeneration, although further investigation is required to unveil the biological roles of laryngeal MSC-like cells in development and homeostasis of tissues.

## Additional file

Additional file 1: Statistical data on relevant means and standard deviations thoughout the experiment. (XLSX 14 kb)

## Abbreviations

BM-MSC: Bone marrow MSC
DMEM: Dulbecco’s Modified Eagle Medium
ECM: Extracellular matrix
EGF: Epidermal growth factor
FBS: Fetal bovine serum
GDF3: Growth differentiation factor 3
GFAP: Glial fibrillary acidic protein
HGF: Hepatocyte growth factor
LP: Lamina propria
MF: Macula flava
MSC: Mesenchymal stromal cell
qRT-PCR: Quantitative reverse transcription-polymerase chain reaction
VEGF: Vascular endothelial growth factor
VF: Vocal fold
